# Causation Analysis of Hazardous Material Road Transportation Accidents Based on the Ordered Logit Regression Model

**DOI:** 10.3390/ijerph17041259

**Published:** 2020-02-15

**Authors:** Changxi Ma, Jibiao Zhou, Dong Yang

**Affiliations:** 1 School of Traffic and Transportation, Lanzhou Jiaotong University, Lanzhou 730070, China; 0217036@stu.lzjtu.edu.cn; 2 College of Transportation Engineering, Tongji University, Shanghai 200082, China; 3 Intelligent Transport System (ITS) R & D Center, Shanghai Urban Construction Design and Research Institute (Group) Co., Ltd., Shanghai 200082, China

**Keywords:** traffic safety, causation analysis, ordered logit model, influence factors, hazardous materials, transportation accident

## Abstract

Understanding the influence factors and related causation of hazardous materials can improve hazardous materials drivers’ safety awareness and help traffic professionals to develop effective countermeasures. This study investigates the statistical distribution characteristics, such as types of hazardous materials transportation accidents, driver properties, vehicle properties, environmental properties, road properties. In total, 343 data regarding hazardous materials accidents were collected from the chemical accident information network of China. An ordered logit regression (OLR) model is proposed to account for the unobserved heterogeneity across observations. Four independent variables, such as hazardous materials drivers’ properties, vehicle properties, environmental properties, and road properties are employed based on the OLR model, an ordered multinomial logistic regression (MLR) is estimated the OLR model parameters. Both parameter estimates and odds ratio (OR) are employed to interpret the impact of influence factors on the severity of hazardous materials accidents. The model estimation results show that 10 factors such as violations, unsafe driving behaviors, vehicle faults, and so on are closely related to accidents severity of hazardous materials transportation. Furthermore, three enforcement countermeasures are proposed to prevent accidents when transporting hazardous materials.

## 1. Introduction

Hazardous materials refer to the substances and articles with explosive, inflammable, toxic, infectious, corrosive, radioactive and other dangerous characteristics that are easy to cause casualties, economic losses or environmental pollution and need special protection during transportation, storage, production, operation, use and disposal [1,2]. Due to the particularity of hazardous materials, the transport of hazardous materials requires professional transport vehicles and corresponding safety protection measures, drivers with professional knowledge and skilled driving skills and specialized personnel to accompany the drivers to transport hazardous materials [3], such as safety training, speed-limit control, GPS position management, regularly check, fatigue driving prohibited, strict transportation plan, continuous working time limit, etc., are proposed for the safety of heavy-truck drivers and roadway travelers [4,5,6]. However, in recent years, transport accidents of hazardous materials occur frequently. Although the number of transport accidents of hazardous materials is relatively small, the consequences of accidents are relatively serious and irreversible [7,8]. On 1 March 2014, for example, two hazardous materials vehicles carrying methanol in a rear-end accident in a highway tunnel in Shanxi Province caused a fire due to a methanol leak, and the other two vehicles stranded of dangerous chemicals and 31 vehicles tram are cited, 40 people were killed, 12 people were injured, and 42 vehicles were burned out. The reason is that the nature of hazardous materials is different, and the hidden dangers of traffic accidents left by the vehicle personnel when they are not filled with the calibration medium when loading and leaving the factory, which causes a deflagration accident when the vehicle collides.

Hazardous materials vehicles will cause huge casualties and economic losses, especially in densely populated residential areas, urban roads, or traffic accidents with passenger cars carrying passengers [8]. Due to the characteristics of radioactive, toxic and dispersing hazardous materials, a large number of residents around the accident site must be removed in time after a traffic accident, and even the soil or air environment around the accident site will be affected. In addition, in the above traffic accidents, the cause of the accident was some personal factors of the driver of the hazardous materials vehicle (such as overload, negligence, failure to operate according to regulations, etc.), modification of the packaging of hazardous materials, and the road chosen (such as tunnels, bridges, etc.) and wet roads caused by rain or snow and other reasons. Therefore, traffic accidents involving hazardous materials vehicles are not only related to the characteristics of the hazardous materials themselves, but also closely related to the attributes of the driver, the selected transportation vehicle, the road environment, and weather conditions [9,10]. Hence, the contributions of our work are to explore the influence factors and related causation of hazardous materials road transportation accidents. This study investigates the statistical distribution characteristics, such as driver properties, vehicle properties, environmental properties, and road properties. In total, 343 data regarding hazardous materials accidents were collected from the chemical accident information network of China. An ordered logit regression (OLR) model is proposed to account for the unobserved heterogeneity across observations. We find that 10 factors such as violations, unsafe driving behaviors, vehicle faults, and so on are closely related to accidents severity of hazardous materials transportation. Furthermore, three enforcement countermeasures are proposed to prevent the transportation accidents of hazardous materials. 

The remainder of this paper is organized as follows. Section 2 reviews the studies on hazardous materials transportation accident. Section 3 lists the data sources in detail, and analyzes the accident distribution characteristics with seven parts. Section 4 describes the factors influencing the severity of road hazardous materials transportation accidents using 343 samples. Section 5 finds there are 10 influencing factors that have a significant effect on the severity of hazardous materials transportation accidents. Section 6 draws the main conclusions and makes a discussion on the key results obtained from the model.

## 2. Literature Review

### 2.1. Accident Data Analysis and Modeling

Hazardous materials transportation accidents are complex events that involve a variety of human responses to external stimuli, as well as complex interactions between the vehicle, roadway features/condition, traffic-related factors, and environmental conditions [11]. The accident data analysis method plays an import role in modeling of accident analysis and prevention. For example, List et al. [12] surveyed research on hazardous materials transportation in the areas of risk analysis, routing/scheduling and facility location, the risk analysis with routing, and routing with facility location were integrated. Van Raemdonck et al. [13] proposed a framework to visualize risk of hazmat transport along a route; they also calculated the local probability of the occurrence of a hazmat transport accident. 

In addition, there are complexities involved in accident analysis and prevention, especially related to vehicle design, age, skill, and hazard perception in driving, impact angles, the physiological characteristics of involved humans, and other factors. For instance, Borowsky et al. [14] examined the effects of age and driving experience on the ability to detect hazards while driving. Ohtani and Kobayashi [15] conducted a statistical analysis of the accident cases of hazardous materials to clarify the cause mechanism of the accident and prevent similar accidents in the future. They found that the reason for the increase of the number of accidents is the uneven and unreliable quality of data cases. Therefore, the reconstructed hazardous materials accident case database ensures the accuracy of cause analysis through multivariate analysis, and makes statistical techniques widely used, such as full Bayesian multivariate count data model [10,16,17], multivariate spatial models [18], multiobjective optimization model [19]. The above accident data analysis and modeling methods make it possible to estimate the risks of hazmat transport along a specific route for transport by road, rail, inland navigation and even pipelines.

### 2.2. Risk Assessment Related the Transport of Hazardous Materials

One of the most important risks in the road transportation of hazardous materials is truck accidents [20,21,22,23], especially collision accidents, in which the main influential factors are the truck drivers’ properties, type of truck, the truck’s safety conditions, environmental properties, and road properties. For example, Shen et al. [20] found that human-related errors (73.8%) and vehicle-related defects (19.6%) were the primary reasons for hazmat tanker crashes, and hazmat tanker accidents mainly occurred in eastern (38.1%) and southwest China (12.3%). Similar results were also found and proven by [21,22,23,24,25].

For the risk assessment related the transport of hazardous materials, some methods have also emerged in the field of risk simulated technologies, GIS-based technologies, job hazard analysis (JHA) method, or statistical model [26,27,28,29,30,31,32,33]. For example, Yu et al. [30] simulated the impact of the oil tank truck accident on the urban environment. They found that the total area affected by pollutants with concentrations above the threshold level may exceed two square kilometers, covering an area where about 5000 people are threatened by dangerous gases of tank accidents. Tixier et al. [31] developed outilde simulation des risques (OSIRIS), a risk simulator, to study the risk simulation of firefighters’ intervention in hazardous materials transportation, the risk simulator OSIRIS can simulate many technical accident cases of different types of accidents. Brzozowska et al. [32] studied and developed a GIS-based emergency response system for major hazardous chemical accidents on highways in order to improve the emergency response efficiency of major hazardous chemical accidents on highways. Ghaleh et al. [33] used the job hazard analysis (JHA) method to identify safety risk contributing factors of road oil trucks. Unlike risk simulated technologies and GIS-based technologies, statistical methods also play an important role in risk assessment, such as statistical analysis of highway accident data [11], statistical analysis of dangerous goods accidents [15], statistical investigation for the characteristics of hazardous chemical accidents in China [17], as well as Bayesian statistical analysis [10,16,34], ordered probit model [35], and probability statistical analysis [36].

### 2.3. Safety Measures Related to Reducing Accident Risk

The safety situation of China’s hazardous materials transportation is grim [15,16,17,18,20,25,28,30,34,37,38,39,40]. Such accidents not only have high spill percentages and consistently large spills, but they can also cause serious consequences such as fires and explosions. Hence, many safety measures related to transport of hazardous materials, such as improving the training of truck drivers, enhancing the quality of vehicles, deploying roll stability aids, enhancing vehicle inspection and maintenance, and developing good delivery schedules [20,23,30,31,32,33,34,35,41,42], are considered to reduce the hazardous material road transportation accidents risk, especially severe crashes.

For example, Noureddine et al. [43] proposed a route planning for hazardous materials transportation using multicriteria decision making method, the similar route planning measures were also proposed by Ma, et al. [1,7,8]. In addition, quality function deployment (QFD) framework [44], risk management framework [45], and security barriers [46], are also considered to manage the local, regional and national transport safety of hazardous materials. Furthermore, among the analysis factors [1,20,22,47,48,49], such as road type, road condition, collision type, age, registration condition and so on, some related safety measures were also proposed [39,50], such as, (a) implement public policies to improve the transport chain of hazardous materials and (b) improve the safety awareness of hazardous materials drivers and observe discipline and law. 

In general, previous studies have done lots of work on hazardous materials transportation accidents, and the research results are various, especially in the analysis of the cause mechanism and countermeasures of hazardous materials transportation accidents, the construction of prediction models for hazardous materials transportation accidents, and risk assessment. However, the results of the study are relatively single, and the underlying laws of transport accidents involving hazardous materials vehicles have not been explored based on accident data. In view of this, taking the hazardous materials vehicle transportation accident data as the research object, through processing the collected hazardous materials transportation accident data, our study is carried out from the hazardous materials transportation accident data statistics, the transportation accident significant influence factor analysis, the accident law discovery, etc. 

## 3. Data Sources and Accident Distribution Characteristics

### 3.1. Data Source 

At present, in addition to the accident statistics of public security departments at all levels and the general analysis of annual accident reports, the data sources for the study of road hazardous materials transportation accidents in China are detailed from the Chemical Accident Information Network. The data included are accident information on road hazardous materials transportation accidents at home and abroad. Traffic accidents are mainly described from the time of the accident, the location of the accident, the weather conditions on the day of the accident, the types of vehicles involved in the accident, the overall overview of the accident, the explanation of the cause of the accident, the number of casualties, and the economic losses caused by the accident. The accident data covers a wide range and a large sample size. 

According to statistics, a total of 343 road traffic accidents involving hazardous materials in China were collected between April 2018 and May 2019. The data used in our study are from the Ministry of Emergency Management (MEM) of the People’s Republic of China. These data are official and authoritative. Before the calculation, we have eliminated the data that do not meet the requirements, mainly including general cargo transportation accidents and hazardous materials explosion accidents in chemical plants.

These data are the latest data that can be collected from the official channels up to now. It is hoped that through these latest data of hazardous materials of transportation, the current safety situation of hazardous materials transportation will be revealed. The research results can provide reference for the construction of a perfect accident prevention system for the hazardous materials supervision department. There were 46 fatalities, accounting for 13.7 percent of the total, and 116 fatalities, accounting for 34 percent of the total. Among them, 19 accidents caused great economic and property losses.

### 3.2. Analysis of Statistical Distribution Characteristics of Road Traffic Accidents of Hazardous Materials

By analyzing the statistical distribution characteristics of the hazardous materials transportation accident data, the overall characteristics of hazardous materials vehicle transportation accidents in various environments, the various influencing factors of transportation accidents, and the relationship between the factors and the overall distribution of the accidents are clarified, and the potential laws of hazardous materials vehicle transportation accidents are quantitatively analyzed from macroscopic perception, and significant factors affecting the severity of transportation accidents are analyzed and accidental factors are eliminated. This article takes the data of 343 hazardous materials transportation accidents as the research object, and statistics the hazardous materials transportation accident data from the aspects such as the cause and type of the accident, driver’s personal and behavioral attributes, road conditions, environmental characteristics, space-time distribution, and cargo attributes analysis.

#### 3.2.1. Types Distribution of Transport Accidents

(1) Causes of transportation accidents

**Figure 1:**
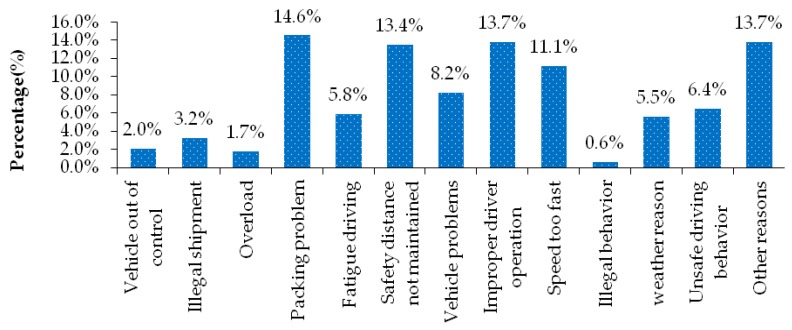
Percentage of the causes of hazardous materials transportation accidents.

Due to a series of factors, such as the special properties of the goods carried, the increasing demand for transport, and the long driving time of drivers, the transport accidents of hazardous materials vehicles become the "flow hazard source" in the road traffic environment. Figure 1 shows the distribution of the cause of the accident of hazardous materials transportation accident. From the Figure 1, we can see, in the distribution of the cause of the accident, the hazardous materials packing problem, driver’s improper operation, safety distance and other reasons is the main cause of hazardous materials transportation accident, respectively accounting for 14.6%, 13.7%, 13.4% and 13.7% of the total number of transportation accident samples. In addition, the number of hazardous materials transportation accidents caused by excessive driving speed, vehicle problems, unsafe driving behavior of drivers and fatigue driving is also relatively large.

(2) Types of transport accidents

**Figure 2:**
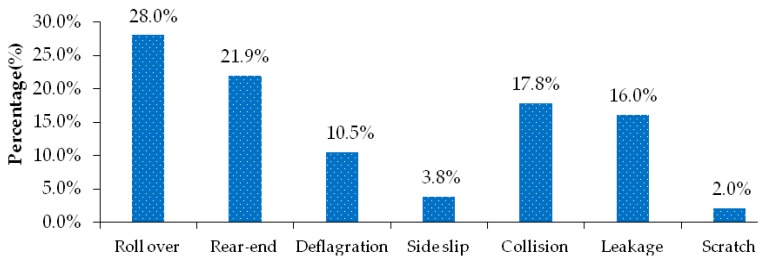
Distribution characteristics of types of hazardous materials transportation accidents.

There are 7 main types of transport accidents of hazardous materials vehicles such as rollaway, rear-end collision, deflagration, sideslip, collision, leakage and scratch. Figure 2 shows the distribution characteristics of hazardous materials and transportation accident types. From the results, it can be seen that roll over is the main accident form of hazardous materials transportation accidents, accounting for 28.0%, followed by rear-end, collision, leakage, deflation, sideslip and scratch, among which rear-end, collision and leakage account for 21.9%, 17.8% and 16.0% respectively.

#### 3.2.2. Month Distribution of Transportation Accidents

**Figure 3:**
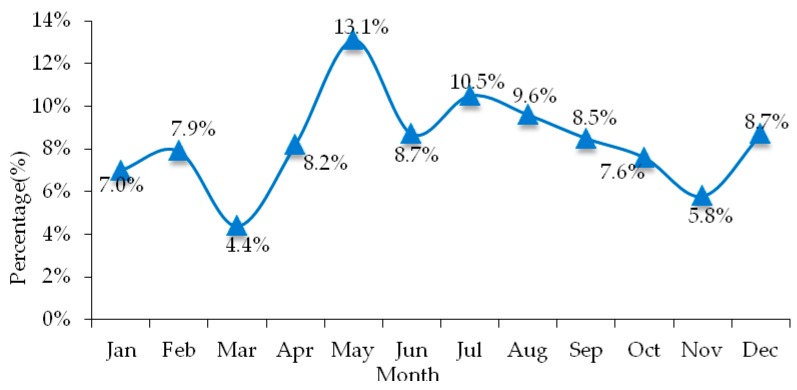
Month distribution characteristics of road hazardous materials accidents.

For the same reason, statistical analysis is performed on the month in which hazardous materials transportation accidents occur, as shown in Figure 3. The results showed that the largest percentage of road hazardous materials transportation accidents were often occurred in May, the value of which was 13.1%, compared with the lowest percentage between 4.4 % and 5.8% in March and November. In addition, the other larger percentage of road hazardous materials transportation accidents were occurred in July and August, the value of which were 10.5% and 9.6%. From Figure 3, it was found that seasonal changes are closely related to hazardous materials transportation accidents, these were, while the temperature was higher (such as May), the percentage road hazardous materials transportation accidents were higher than other seasons; while the temperature was lower (such as November), the percentage road hazardous materials transportation accidents were lower than other seasons. The reason was that when the temperature was high, the vehicle ran for a long time, which caused the tires to heat up, and the temperature of hazardous materials transported in the tank raised, accidents such as collisions, rollovers, and rear-end collisions were prone to occur. Therefore, during May-July, in the transportation process of hazardous materials, the temperature of the oil tank must be monitored at all times and effective control should be performed to avoid accidents.

#### 3.2.3. Time Distribution of Transportation Accidents

**Figure 4:**
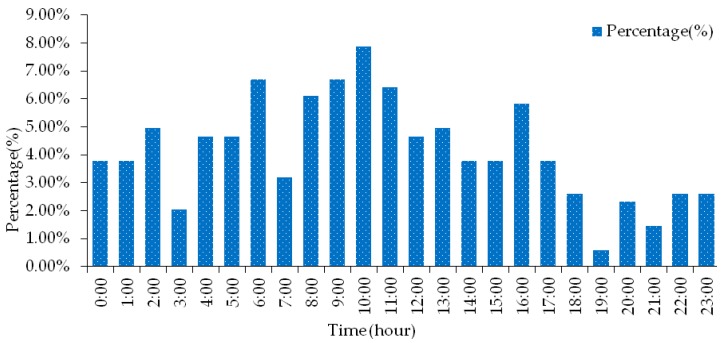
Time distribution characteristics of road hazardous materials transportation accidents.

The statistical analysis of the time point of road hazardous materials transportation accident is shown in Figure 4. It can be seen from the graph that the largest number of road hazardous materials transportation accidents in a day is at 10 o’clock, accounting for 7.87% of the total number of accidents, and it was found that at 2 am, 4–6 am, 8–13 am, and 16 o’clock, The number of accidents in this time period was higher than the average number of accidents per hour. The total number of accidents accounted for 63.57% of the total, indicating that this time period is a high-frequency period for accidents involving hazardous materials vehicles. At 6:00 o’clock, most of the time is when urban roads are open to the outside, allowing hazardous materials vehicles to pass through and entering the city. Due to poor visibility of drivers at night, and in order to shorten transportation time, more drivers will fatigue driving, likely to cause traffic accidents. There are many accidents between 8–13 and 16, because this time period is the highest temperature of the day (especially in summer), when the hazardous materials transported when the tank is heated and the temperature is high, it is easy to cause hazardous materials to deflagrate, or when the surface temperature is too high, when the hazardous materials vehicle is driven for a long time, the tire friction and heat will cause a deflagration accident. In addition, the number of vehicles in this period is relatively large, and it is more likely to cause hazardous materials transportation accidents when the road traffic environment is more complicated.

#### 3.2.4. Regional Distribution of Transportation Accidents

**Figure 5:**
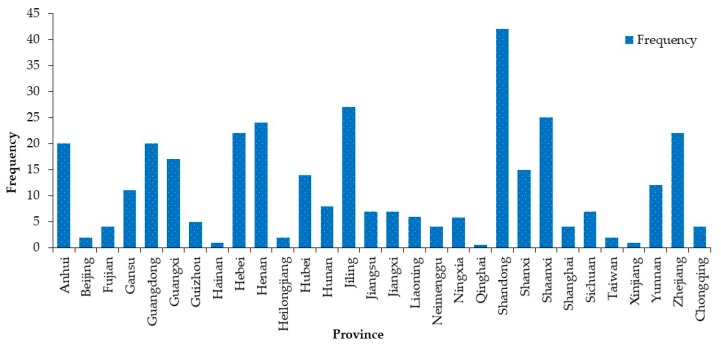
Regional distribution characteristics of road hazardous materials transportation accidents.

Statistics are made on the provinces where road hazardous materials transportation accidents occur, and the results are shown in Figure 5. As can be seen from Figure 5, Shandong Province has the largest number of road hazardous materials transportation accidents, accounting for 12.24% of the total number of accident samples, followed by Shanxi Province, Jiangsu Province, Henan Province and Zhejiang Province, accounting for 7.29%, 7.87%, 7.00% and 6.41%, separately. The total number of accidents of the five provinces accounted for more than 40.81%. Therefore, for the provinces where transportation accidents occurred frequently, some measures such as renovating road infrastructure, promoting driver’s publicity and education, supervision, and early warning, et. al, needed to be proposed by the road administrative departments, so as to reduce the impact of hazardous materials on the environment along the road.

#### 3.2.5. Vehicle Category Distribution of Transportation Accidents

**Figure 6:**
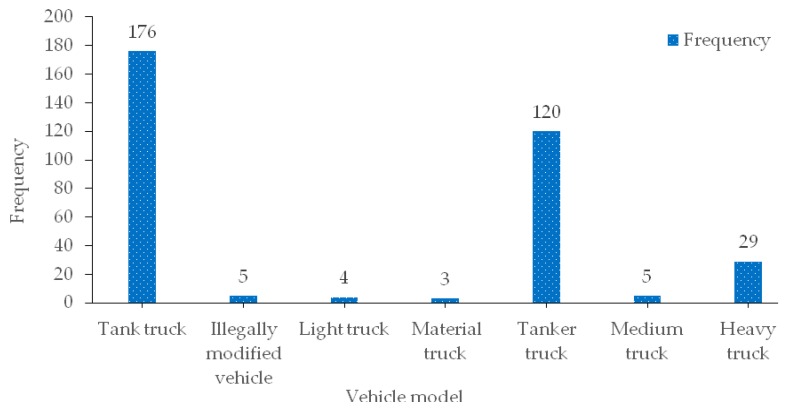
Vehicle models distribution characteristics of road hazardous materials transportation accidents.

Due to the limitation of the producing area of hazardous materials and the increase of the demand in various places, the transport volume of hazardous materials in a single trip is relatively large. Therefore, the hazardous materials transportation vehicles selected during transportation are basically large or heavy vehicles, mainly tank trucks (such as oil tank trucks, tank trucks that do not carry oil, etc.) and trucks of different tonnage (such as van, light, medium and heavy open trucks, etc.). This paper makes statistics on the vehicles involved in the accident, as shown in Figure 6. 

The statistical results show that among the vehicles involved in the road hazardous materials transportation accident, tank trucks account for 86.6%, of which 35.1% are oil tank trucks (carrying gasoline or diesel), and 51.5% are other hazardous materials (carrying methanol, acetic acid and other hazardous materials). Because it is difficult to protect the safety of the tank and control the properties of the tank during transportation, it is difficult to ensure the integrity of the tank and the stability of the hazardous materials in the tank when accidents occur. Therefore, once a transportation accident involving tank trucks occurs, it may cause more serious accident consequences.

In addition, in the data analysis, it was found that some of the accident vehicles involving hazardous materials were illegally modified or carried in violation of regulations, accounting for 1.5% of the overall proportion. Although the proportion is small, compared to fully equipped professional vehicles, in other words, the potential safety risks and social interference in such vehicles are even more serious. Therefore, non-exclusive vehicles must be strictly prohibited from transporting hazardous materials on the road.

#### 3.2.6. Road Distribution of Transportation Accidents

**Figure 7:**
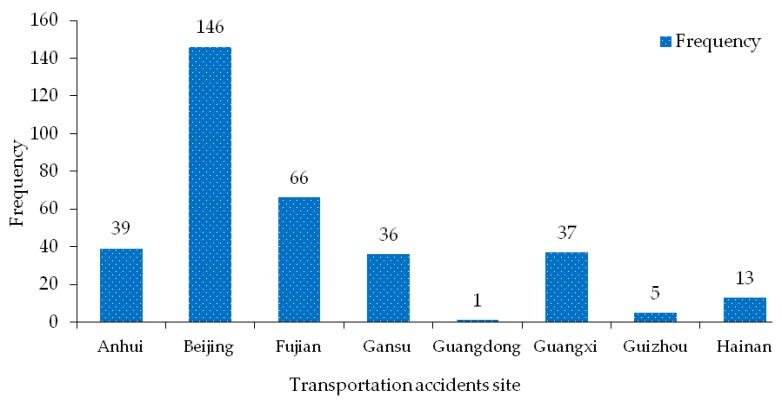
Road distribution characteristics of hazardous materials transportation accidents.

The driving environment and speed requirements of hazardous materials vehicles will be different if drivers choose different driving roads. According to the statistics of the road types where hazardous materials transportation accidents occur, the statistical results are shown in Figure 7. 

The result shows that the most hazardous materials transportation accident road is highway, which account for 42.57% of the total number of accidents, It can be seen that the highway is the most prone to traffic accidents for hazardous materials vehicles, which is related to the range of driving speed. Once these situations occur while driving, such as driving for too long causes the tire to heat up, the distance from the car is too close, the safety clearance left when passing is too small, the driver in fatigue driving, and the speed at the curve is too fast and over-speed, poor tank sealing or loose valves, etc., it will cause a traffic accident with hazardous materials, and it will be accompanied by more serious traffic accident consequences. Therefore, drivers and escorts of hazardous materials must maintain correct driving safety awareness and standard management concept of hazardous materials to ensure the safety of hazardous materials transportation on the highway. In addition to expressways, national highways, urban roads, provincial highways and intersections are also frequent types of hazardous materials transportation accidents, accounting for 51.9% of the total accident data due to the constraints of traffic infrastructure and traffic conditions. The accident sites of these types of roads are mainly located in the up-slope curves, down-slope curves, ramp crossings and intersections, which are the strongholds or nodes of road passage, and the nodes with more interference of vehicle flow intersection and restricted road passage conditions. When the vehicles do not slow down at the intersection, the speed is too fast at the curve, and they are not familiar with the road ramp marks, traffic accidents are likely to occur in the transportation of hazardous materials. Furthermore, if there is a traffic accident with hazardous materials on urban roads and intersections, the leakage of hazardous materials will pose a great threat to the lives of residents and urban infrastructure on both sides of the road or near the intersection. Therefore, it is necessary to reduce this type of traffic through research and guarantee the safety of life and property of residents along the road.

#### 3.2.7. Weather Distribution of Transportation Accidents

**Figure 8:**
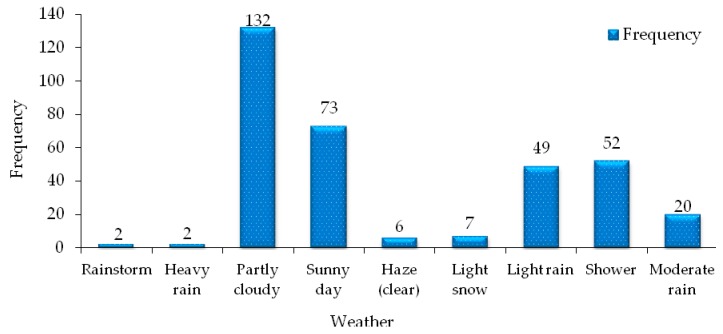
Weather distribution characteristics of hazardous materials transportation accident.

The weather on the driver’s driving day has a certain influence on his driving safety. The weather distribution at the time of road traffic accidents of hazardous materials is statistically analyzed, as shown in Figure 8. As can be seen from the statistical data in the Figure 8, cloudy weather is the most frequent weather on the day of the road hazardous materials transportation accident, accounting for 38.48% of the total number of accidents. In addition, rainy days and snowy days also account for 38.48% of the total number of accidents. It can be seen that in the transport accidents of hazardous materials vehicles, there are much rainy or snowy days. Because when the day chosen to travel is rainy and snowy, the road is slippery and the driver’s vision is blocked, nearly half of the hazardous materials vehicles will choose the expressway to travel, and the driving speed is too fast, when they are too close to the car or improper operation will occur, hazardous materials transportation accidents are prone to occur. Therefore, avoiding driving in rainy and snowy weather is a way to reduce the occurrence of hazardous materials transportation accidents on the road. In Shanxi province, during the transportation of hazardous materials, fog and haze (sunny days) occurred, accounting for 1.75%. When drivers drive at high speed on expressways, their visibility is severely restricted, so traffic accidents are more likely to occur in such a low guarantee environment. When the traffic flow in the driving environment is large, once a hazardous materials transportation accident occurs, it will cause greater casualties and property losses. Therefore, relevant management departments should prohibit hazardous materials drivers to drive in the haze weather, in order to prevent traffic accidents caused by this kind of weather.

## 4. Analysis of Factors Influencing the Severity of Road Hazardous Materials Transportation Accidents

There is a certain relationship between the severity of road hazardous materials transportation accidents and the influencing factors. Establishing the relationship between the influencing factors and the severity of hazardous materials transportation accidents through statistical methods is an important method to determine the influencing factors of the severity of traffic accidents and study the law of road hazardous materials transportation accidents. The severity of hazardous materials transportation accidents can be regarded as a discrete order problem from the perspective of mining the law of transportation accidents. The traditional discrete selection model cannot reflect the performance of the severity of the accident, but the multi-classification ordered Logistic regression analysis provides a good solution for such data.

Therefore, this section is based on the collection and compilation of data samples of 343 hazardous materials transportation accidents on the road. First, the possible influencing factors of hazardous materials transportation accidents are identified through single factor analysis, and the selected influencing factors are subjected to a co-linearity test. In the factor analysis, *p* < 0.1 and no co-linearity factors were included in the multi-factor ordered logistic regression model to determine the significant influencing factors of hazardous materials transportation accident levels. Finally, the elasticity analysis was introduced to quantitatively identify the significant influencing factors of road hazardous materials transportation accidents, provide the basis for the accident rule mining.

### 4.1. Data Preparation

The severity of traffic accidents is divided into four levels: death accident, serious injury accident, minor injury accident and property loss accident [22]. However, according to the statistical processing results of road hazardous materials transportation accident information, it is known that because the existing statistics on hazardous materials transportation accident information that can be collected are not detailed enough, it is impossible to specifically distinguish serious injuries from minor injuries in the processing of accident data. So serious injuries and minor injuries are classified into one level. Therefore, on the premise of comprehensive consideration of death, injury and property damage in transportation accidents, the severity of road hazardous materials transportation accidents can be divided into three categories: Y=1 deaths, Y=2 injuries, and Y=3 property damage. The analysis of the factors influencing the severity of hazardous materials transportation accidents is carried out by using these three levels as the dependent variables of the prediction model.

### 4.2. Analysis of Influencing Factors

The selection of the independent variables in our study are driver properties, vehicle properties, environmental properties, road properties. These independent variables come from the factual evidence of hazardous material road transportation accidents in China as well as the findings from statistical distribution characteristics of road hazardous transportation accidents. In addition, some independent variables in our study are come from Refs. [1,3,20,22,24,25,28,31,34].

A total of 44 sub-variables were included in the statistical processing results of the accident data. When selecting the influencing factors of road hazardous transportation accidents, the research results of hazardous materials transportation accidents were referred to, and some of the useless information was removed by collating the collected data. According to the analysis of the statistical distribution characteristics of road hazardous transportation accidents, and the actual distribution of each type of data in the survey data, some independent variables are coded, deleted, merged, and grouped. The independent variable set including behavior attributes of the hazardous materials driver, vehicle attributes, road attributes, environmental attributes was reconstructed. The variable set contains 11 variables. The specific information is shown in Table 1.

In addition, in the study of traffic accidents, “collision type” (frontal collision, side collision, etc.) is often used as an influencing factor for traffic accident prediction, thereby greatly improving the accuracy of model prediction. However, some studies have pointed out that the type of collision is the result of the effects of other accident factors, and there is a logical cause–effect contradiction when take it as an independent variable. Therefore, the independent variables selected in this paper do not include the collision type factor.

### 4.3. Univariate Analysis of the Influence on the Severity of the Accident

Before the logistic regression analysis, the single factor analysis was used to identify whether the selected possible influencing factors had significant differences in the mean level of the severity of hazardous materials vehicle transportation accidents. The rank-sum test is a non-parametric test method, which is suitable for the difference analysis of grade data. The hazardous materials transportation accident grade is a typical grade data. Therefore, the rank-sum test is used to carry out the single-factor test for 11 influencing factors.

The proportion of death, injury and property loss accidents in 343 road hazardous materials transportation accidents was 13.12%, 33.53% and 100%, respectively. Factors that may affect the severity of transportation accidents of hazardous materials were analyzed by rank-sum test. The analysis results are shown in Table 2. The analysis results showed that there were statistically significant differences in severity composition of 11 influencing factors of hazardous materials transportation accidents (*p* < 0.05). Therefore, it can be considered that the 11 influencing factors selected can be used for the ordered logistic regression analysis of the severity of hazardous materials transportation accidents.

### 4.4. Parameter Calibration and Elastic Analysis

In order to identify the significant influencing factors that affect the severity of hazardous materials transportation accidents, 11 influencing factors including violations, unsafe driving behaviors, and accident liability are taken as independent variables, and the severity of the accident is used as the dependent variable. An ordered Logit model is used to establish an estimated relationship between the severity of the accident and the influencing factors, and use the maximum likelihood estimation to perform parameter calibration of the model [23,24,25,26,27].

Accident severity (dependent variable) (*y*) is 3 ordered discrete variables, the unobservable dependent variable y* is the latent variable corresponding to accident severity (*y*), *X* is 11 independent variables such as illegal behavior and unsafe driving behavior,β is the corresponding parameter to be estimated, and u is the error term subject to Logistic distribution.

Let $\alpha_{1}<\alpha_{2}<\alpha_{3}$ represents the estimated critical value, and the relationship between $y^{*}$ and *y* depends on whether it is greater than or less than the given critical value, that is:

If $y^{*}\leq \alpha_{1}$, is the economic loss accident, *y* = 1; If $\alpha_{1}<y^{*}\leq \alpha_{2}$, is an injury accident, *y* = 2; If $\alpha_{2}<y^{*}$, is a fatal accident, *y* is equal to 3.

Among them,

(1) $$y^{*}=X\beta +u$$

Thus, given the independent variable *x*, the response probability of the dependent variable *Y* at each value can be calculated, namely:

(2) $$\left\{ \begin{array}{l} p(y=1)=p(y^{*}\leq \alpha_{1})=p(X\beta +u\leq \alpha_{1})=\phi (\alpha_{1}-X\beta )\\ p(y=2)=p(\alpha_{1}<y^{*}\leq \alpha_{2})=\phi (\alpha_{2}-X\beta )-\phi (\alpha_{1}-X\beta )\\ p(y=3)=p(y^{*}>\alpha_{2})=1-\phi (\alpha_{3}-X\beta ) \end{array}\right.$$

Ordinal multiple classification logistic regression was used to calibrate the parameters. According to the significance test of the independent variables, the method of stepwise regression is adopted to screen the independent variables to check whether the introduction of the selected independent variables into the model has a significant change in the prediction results of the model.

The model likelihood ratio test showed that the ordered Logit regression model was meaningful ($\chi^{2}$ = 215.327, *p* < 0.001). The goodness of fit test showed that the model fitted well (Pearson’s test *p* = 0.327), indicating that the model fitted well. The results showed that the data met the presupposition of proportional advantage ($\chi^{2}$ = 5.396, *p* = 0.249 > 0.05), indicating that the data was suitable to be analyzed by the ordered Iogit model. The model fitting results are shown in Table 3.

To determine whether a variable has a significant impact on the severity of hazardous materials transportation accidents, Wald statistic test can be used. The greater the value of Wald $\chi^{2}$ or the smaller the significant probability, the greater the contribution of the independent variable. The critical value of significance probability of variables is 0.05. According to the contribution rate of influencing factors, it is found that 10 independent variables have significant influence on the severity of hazardous materials transportation accidents.

Significant factors include illegal behavior, unsafe driving behavior, accident liability, vehicle problems, vehicle class, weather, lighting, seasonal distribution, road grade, and regional distribution. However, the critical value of significance probability of packaging and other variables was greater than 0.05, and it was considered that the influence on the severity of road hazardous materials transportation accidents was not significant.

In Table 3, the cumulative odds ratio (OR) value reflects the effect of the influencing factors of hazardous materials on the severity of transport accidents. If the OR value is greater than 1 in the data calibration, it indicates that the possibility of serious accidents of road hazardous materials increases under the influence of this sub-independent variable. On the contrary, if the OR value is less than 1, it indicates that the possibility of serious accidents of hazardous materials increases OR decreases under the influence of the independent variable.

Although the influence of significant factors on the severity of the accident can be qualitatively explained according to the formula of ordered Logit model and the regression coefficient value of the established model, it cannot quantitatively explain how each factor affects the probability of the occurrence of the severity of the accident. To solve this problem, the concept of elasticity analysis [22] is introduced as a marginal effect evaluation to measure the impact of independent variable changes on the probability of accident severity. For classification variables, pseudo-elasticity $E_{X_{nk}}^{P_{in}}$ can be calculated according to the following formula:

(3) $$E_{X_{nk}}^{P_{in}}=\frac{P_{in}[givenX_{nk}=1]-P_{in}[givenX_{nk}=0]}{P_{in}[givenX_{nk}=0]}$$

The pseudo-elasticity value can be used to calculate the percentage change in the probability $P_{in}$ of accident severity i when the individual n’s first k independent variable $X_{nk}$ changes from 0 to 1.

As for the classification of accident severity, the pseudo-elastic value of a given independent variable is the percentage change in the probability of occurrence of each individual n, so the pseudo-elastic value can be calculated with the mean value of all observed objects. The results of elasticity analysis for 10 significant influencing factors with *p* value less than 0.05 in the ordered multi-classification Logistic regression analysis are shown in Table 4.

## 5. Analysis of Factors Affecting Accident Severity

Assume that the constant term of the property loss accident in the model is 0, and the constant term in the utility function can reflect the potential serious accident probability without considering any possible influence factors. As shown in Table 3, the estimated values of the constant terms of the death and injury accidents in road hazardous materials transportation accidents are −1.461 and 1.790 respectively, which shows that without considering the influencing factors of other hazardous materials transportation accidents, at the moment of the driver’s decision, the driver is more likely to be injured (or more likely to be harmless) rather than fatal. According to the calibration and fitting results of the model, it can be seen that there are 10 influencing factors that have a significant effect on the severity of hazardous materials transportation accidents. Then, the elasticity analysis of the influencing factors is performed based on the pseudo-elastic value.

### 5.1. Behavior Attributes Analysis of Hazardous Materials Driver

(1) Violation of regulations

It can be seen from Table 3 that the regression coefficients of illegal overload and fatigue driving are −1.384 and −1.934, respectively, and the OR values are 1.41 and 1.20, respectively, indicating that in the case of hazardous materials transportation accidents, the results of accidents are more likely to be serious when the driver of hazardous materials violates the law of overload and fatigue driving than when the driver violates the law of carriage.

It can also be seen from the above data table that the hazardous materials driver has a higher probability of serious transportation accidents when driving illegally and overloading, and fatigue driving. Elasticity analysis result shows that the violations of hazardous materials overload and fatigue driving, illegal behavior of death accident possibility are increased by 44% and 113.8% respectively. Among them, the elasticity corresponding to the violation of the dangerous driving behavior caused by fatigue driving of the driver exceeded 1.000, indicating that the influencing factor has a significant impact on road hazardous materials transportation accidents. It can be seen that fatigued driving should be listed as one of the illegal acts strictly prohibited in the transportation of hazardous materials drivers on the road, because fatigued driving will increase the probability of hazardous materials transportation accidents, especially fatal accidents, based on the analysis of fitting data and elasticity analysis results. The possibility of an accident will increase significantly under the influence of this factor.

(2) Unsafe driving behavior

According to Table 3, the regression coefficients of the hazardous materials driver’s unsafe driving behavior such as fail to maintain a safe distance and excessive speed are −1.435 and −1.236, respectively, and the OR values are 1.647 and 1.539 respectively, indicating that the failure to maintain a safe distance and excessively fast vehicle speeds has a greater contribution to the severity of hazardous materials transportation accidents than others, that is, when the driver of a hazardous materials fails to maintain a safe distance and excessively fast vehicles during driving, transportation accidents are more likely to happen.

Similarly, according to the above situation, combined with the elasticity analysis results shown in Table 4, it can be seen that unsafe behavior in hazardous materials drivers includes not maintaining a safe distance and driving too fast. The death rates for these behaviors are 256.8% and 269.3%, which indicates that when a hazardous materials driver has a transportation accident without maintaining a safe distance or too fast, the severity of the accident is more inclined to a fatal accident. Different driving behaviors will increase the possibility of fatal accidents in the transportation of hazardous materials. In addition, the elasticity values of non-concession and excessive speed are both greater than 1.000. Therefore, these two factors have a significant impact on hazardous materials transportation accidents (especially fatal accidents). It can be seen that during the transportation of hazardous materials, the driver (escorted person) must not only maintain a reasonable safe distance from other vehicles running on the road, but also must maintain a safe road speed regardless of whether the road environment is smooth or not. Driving speed should be within the control range.

(3) Liability for accidents

Hazardous materials vehicles are the main participants in road hazardous materials transportation accidents. Of the 343 hazardous materials transportation accidents that occurred during the one-year period, the responsibility for accidents is that hazardous materials vehicles accounted for 82.8% of the total accidents. It can be seen that this type of vehicle is the most responsible for accidents involving hazardous materials vehicles. As can be seen from Table 3, the regression coefficient of the hazardous materials vehicle is −2.429, and the OR value is 2.363, which indicates that compared with other vehicles, the hazardous materials vehicle has a higher contribution to the severity of the accident in hazardous materials transportation accidents. Similarly, by analyzing the data in Table 4, it can be seen that the contribution value of hazardous materials vehicles in the elasticity value analysis is 323.4%, and the value is greater than 1.000, which indicates that the influencing factors significantly increase the probability of fatal accident of the hazardous materials transportation accidents. Furthermore, although this kind of influencing factor does not show a significant impact on injury accidents and property loss accidents, both can increase the severity of these two accidents by 45.7% and 87.3%.

In summary, once a hazardous materials vehicle is involved in a road traffic accident, whether it is a death, injury, or property loss accident, it will cause more serious accident consequences, especially a property loss accident, because most of the hazardous materials vehicles are tank trucks (oil tanker trucks). When such vehicles are involved in traffic accidents, most of them will cause cracks in the tank or damage to the tank valves, which will cause leakage of hazardous materials in the tank. So, even not considering the damage and loss of hazardous materials vehicles, each accident will cause the loss of hazardous materials due to tank damage, which will greatly increase the severity of the property damage.

### 5.2. Vehicle Attributes Analysis of Hazardous Materials Transportation Accident

(1) Packaging issues

From the regression analysis data of the Logit model, it can be seen that the return coefficient values of the independent valve looseness and the tank rupture are −0.597 and −0.798, respectively, and the OR values are 0.302 and 0.450 (the reference object is the tank fire). Its OR value is not greater than 1.000, which indicates that the two influencing factors have not increased the severity of the hazardous materials transportation accident, that is, the possibility of serious accidents in the transportation of hazardous materials is pretty low under the two influence factors. Furthermore, because the Logit regression *p* values of valve looseness and tank rupture are all greater than 0.05, it is considered that the packaging problem (independent variable) has no significant impact on the severity of road hazardous materials transportation accidents, and therefore does not have the conditions for elastic value analysis. However, in reference to the classification of the fire in the tank of the target object, the number of fatal accidents and injuries caused by the packaging problems accounted for 33.3% and 50% of the total accident data caused by the packaging problems. It can be seen that compared with the loose valve and the tank rupture those two independent variables are concerned, the fire in the tank has a greater impact on the severity of hazardous materials transportation accidents.

(2) Vehicle problems

In the transportation of hazardous materials, when the vehicle suffers from tire burst, out of control, brake failure, engine fire and other problems, it will cause more serious property loss accidents, especially the vehicle out of control. Once it occurs, it will cause a serious fatal accident (in the independent variable classification of vehicle problems, 100% of fatal accidents in hazardous materials transportation accidents involve a vehicle out of control) and injuries (50%). As can be seen from Table 3, the regression coefficient of vehicle runaway is −1.096, and the OR value is 1.201, which indicates that compared with other sub-independent variables in the vehicle problem, vehicle runaway has a higher contribution to the severity of hazardous materials transportation accidents. Furthermore, according to the elasticity analysis in Table 4, it can be seen that the probability of fatal accidents when a hazardous materials vehicle gets out of control has increased by 32.40%. It can also be seen that before the hazardous materials driver or escorted person is transported by car, the hazardous materials vehicle must be carefully inspected to avoid as far as possible the influential factors such as the loss of control of hazardous materials vehicles during the driving process, which can cause more serious accident consequences. To ensure the safe delivery of hazardous materials to their destination.

(3) Vehicle category

The severity and probability of accidents caused by different types of vehicles during the transport of hazardous materials vary. According to statistics, tank trucks are the type of hazardous materials vehicles most involved in hazardous materials transportation accidents, accounting for 48.69% of the total number of accidents, followed by oil tankers and trucks, which respectively account for 34.11% and 11.37% of the total number of incidents. It can be seen that tank trucks and oil tank trucks are the most important types of vehicles involved in hazardous materials transportation accidents. Therefore, in order to reduce the probability of hazardous materials transportation accidents, we must pay more attention to the education and driving skills of these two drivers. Train and conduct regular safety inspections before each straight transportation mission to ensure transportation safety.

In the vehicle category, the corresponding OR values of tanker trucks, oil tanker trucks and trucks are 4.942, 3.507, and 1.848, which indicates that for the control variables, these three influencing factors have a high contribution to the severity of the hazardous materials transportation accident. Furthermore, in the elasticity analysis of Table 3, the probability of fatal accidents in tank trucks, oil tank trucks and trucks in hazardous materials transportation accidents increased by 213.40%, 287.60%, and 23.30%, indicating that these three factors can increase the probability of death. Especially for oil tank trucks, fatal accidents accounted for 42.86% of total fatal accidents. Due to the large amount of diesel or gasoline transported by oil tank trucks, once a traffic accident (collision, bump, rear-end collision, rollover accident) occurs, it will cause damage to the carrying tank, crude oil leakage, frictional heating of the tank, etc. In all cases, explosions will occur under certain uncontrollable conditions. Therefore, the casualties and property damage caused are particularly serious. Therefore, the driving of oil tank trucks requires more advanced driving skills and a safe driving environment than other hazardous materials vehicles.

### 5.3. Environmental Attributes Analysis of Hazardous Materials Transportation Accidents

(1) Weather

Weather as an inalterable factor is a more or less essential factor in any traffic accident. Therefore, the occurrence of hazardous materials transportation accidents is no exception without the participation of weather factors. In the classification of weather variables, the regression coefficients of cloudy as well as rainy and snowy weather are −1.813 and −2.346, and the OR values are 1.393 and 2.622, respectively, indicating that cloudy as well as rainy and snowy weather have a higher contribution to the severity of hazardous materials transportation accidents. The elasticity analysis shows that the probability of death of hazardous materials in cloudy and rainy snow weather increased by 31.70% and 155.20% compared to other weather types, which indicates that in the case of rainy and snowy weather, once the traffic of hazardous materials vehicles occurs, The probability of accidents and deaths has increased by nearly 1.5 times, which has a significant impact on fatal accidents in the transport of hazardous materials.

**Figure 9:**
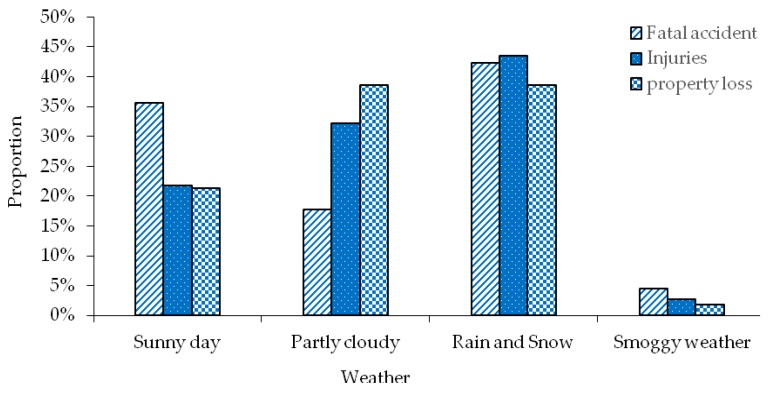
Graph of proportion of traffic accidents with weather sub-independent variables.

It can be seen from the analysis results that although cloudy weather as well as rain and snow are not significant factors affecting hazardous materials transportation accidents, they can enhance the severity of hazardous materials transportation accidents to a certain extent, especially rain and snow weather. In the statistics of the variables, the number of fatal accidents, injuries, and property damages in rainy and snowy weather accounted for 42.22%, 43.48%, and 38.48 of the total number of statistical accidents, as shown in Table 5 and Figure 9. It can be seen that there are more accidents of hazardous materials transportation in rainy and snowy days, and the degree of accident casualties is more serious. Therefore, the driver of hazardous materials or the relevant transportation company needs to deliberately avoid the rainy and snowy weather when choosing the time period for vehicle transportation, and further reduce the occurrence of hazardous materials transportation accidents from the subjective consciousness, and in turn try to eliminate the death of hazardous materials transportation as far as possible from the root of the weather, so as to minimize the loss of transportation accidents and ensure the safety of travel.

(2) Lighting situation (point in time)

In order to reduce the time cost in the transportation process as much as possible, the domestic and foreign transportation units not only drive the hazardous materials during the day but also drive uninterruptedly at night. Most cities only let trucks or hazardous materials pass at night. So traffic accidents have increased to a certain extent. As shown in Table 3, the regression coefficient at night without lighting is −1.08, and the OR value is 1.111, which indicates that compared with the daytime, no lighting at night can increase the severity of hazardous materials transportation accidents, that is, the contribution to the severity of the accident is relatively large. Elasticity analysis shows that no lighting at night can increase the probability of fatal accidents in hazardous materials transportation by 134.6%, which indicates that in the case of no street lighting at night, once a hazardous materials transportation accident occurs, the probability of fatal accidents will be greater than that of injuries. Accidents and property loss accidents increased by about 1.3 times, that is, no lighting at night has a significant impact on the severity of transportation accidents.

According to the analysis results, in the occurrence of hazardous materials transportation accidents involving night no lighting only accounted for 25.07%, and in this proportion of accident statistics, casualties accounted for 50%, indicating that although the probability of an accident that under the condition of no lighting is low, once it occurs, there is a half probability that the accident will become a serious casualty. Therefore, hazardous materials drivers should try not to drive under the condition of no traffic lighting, in order to avoid serious traffic accident which results in irreversible loss, and relevant supervision departments should also increase the supervision and punishment of hazardous materials driving at night and strive to reduce the occurrence of hazardous materials transportation accidents at the source. 

(3) Monthly distribution

Different months of the year are invisible factors that affect the safety of hazardous materials transportation. Due to the special nature of hazardous materials (a certain physical and chemical characteristics change with different temperature and humidity), it has impact on safe transportation of hazardous materials, especially in the second quarter. As can be seen from Table 3, the regression coefficient of this factor is −0.563, and the OR value is 2.399, which indicates that compared with other quarters, this quarter can aggravate the severity of hazardous materials transportation accidents. That is, a greater contribution to the severity of the accident. The elasticity analysis shows that when traffic accidents occurred during the transportation of hazardous materials in the second quarter, the probability of fatal accidents increased by 39.50%, which is related to the weather temperature. Because when a vehicle collides, rolls over, rear-ends, or bumps, a serious explosion accident often occurs or the temperature of the tank itself due to contact between the vehicle tank and other vehicles or the vehicle’s own tank shaking. The probability of a fatal accident, so the driver keeps the safe driving distance as much as possible, and adjusts the tank temperature in the tank as much as possible to ensure the safety of transportation.

### 5.4. Road Attributes Analysis of Hazardous Materials Transportation Accidents

(1) Road grade

Different transportation roads selected by the driver of hazardous materials have different potential safety hazards. The driver is not familiar with the specific road traffic conditions of the selected transportation route, and is unskilled in handling different road types during driving. When an accident occurs, the severity of the accident will be increased to a certain extent, such as provincial roads. Although the participating property loss accidents accounted for 10.79% of the total number of accidents, among them, the number of injuries and fatalities accounted for 62.16% of the total number of property loss accidents. According to the provincial road, the OR value shown in Table 3 is 1.174, showing that provincial roads are dangerous. The contribution of the severity of materials transportation accidents is relatively large.

According to the elasticity analysis in Table 4, it is known that the probability of fatal accidents in the transportation of hazardous materials increased by 81.10%, 42.60%, 31.60%, and 13.20% for highways, national highways, provincial highways, and urban roads (intersections), respectively. It can be seen that the growth probability corresponding to the expressway accounts for 48.13% of the total probability increase, which indicates that when a hazardous materials traffic accident occurs on the expressway, the probability of fatal accidents is higher than other road types. This is because the driving speed on the expressway is fast, when the vehicle is too close to the vehicle, the speed is too fast at the corners, and the road markings are not clear, the driver’s reaction operation time is too short, causing the driver to fail make reasonable operations that can ensure safe driving within the allowed time, and then a more serious hazardous materials transportation accident (death accident) caused by excessive speed, so drivers of hazardous materials must strictly follow the guidelines when driving on the highway, driving safely in a dedicated lane and a reasonable speed zone.

(2) Regional distribution

Nationwide, due to the different natural resource reserves and energy demand, the distribution of hot zones for hazardous materials also varies, so the regional distribution of hazardous materials accidents also varies. According to Logit regression analysis, the regression coefficients of the three regions of North China, East China, and Northwest China are −1.373, −0.473, and −1.161, and the OR values are 1.52, 2.21, and 1.6, which indicates that compared with the northeast region, the severity of hazardous materials transportation accidents in the three regions is higher. The elasticity analysis found that the probability of fatal accidents in the three regions of North China, East China and Northwest China increased by 21.90%, 114.30%, and 26.60%, especially in East China, because the elasticity value of this area is greater than 1.000, indicating that the factor has a significant impact on hazardous materials transportation accidents, especially fatal accidents, and the number of fatal accidents in East China accounts for 42.86% of the total number, which is closely related to the energy storage and energy transportation needs in East China. Therefore, relevant departments should focus on strengthening the supervision of hazardous materials transportation in East China, using more effective safety awareness education and publicity and supervision system to reduce the occurrence of transportation accidents in the region, and minimize the seriousness caused by casualties and property damage.

## 6. Conclusions and Suggestions

### 6.1. Conclusions

Based on 343 hazardous materials transportation accidents, this paper analyzes the statistical distribution characteristics of hazardous materials transportation accident types, drivers and driving behaviors, roads and environmental conditions, and then constructs driver behavior attributes, vehicle attributes, and environment based on the statistical distribution characteristics. The independent variable set of the attributes and road attributes contains a total of 11 variables, then use ordered Logit model to estimate the hazardous materials transportation accident damage to property, injury and death three level transport accident probability, the distribution of quantitative analysis and on the basis of the theory of elastic analysis of the hazardous materials transportation accident severity significantly influence factors. The analysis results show that 10 factors, such as illegal behavior, unsafe driving behavior, accident liability, vehicle problem, type of vehicle, weather, lighting, road grade and regional distribution, are closely related to the severity of road hazardous materials transportation accidents. Specific conclusions are as follows,

(1) The probability of fatal accidents when drivers of hazardous materials have overloaded and fatigued driving has increased by 44% and 113.8% compared with other violations; and the probability of fatal accidents due to failure to maintain a safe distance and excessive speed increased by 2–3 times respectively, and fatal accidents involving hazardous materials involving unsafe driving behavior accounted for 61.9% of the total number of accidents; vehicle problems are also significant factors affecting the severity of transportation accidents, for example, one of the sub-variables-vehicle out of control makes roads dangerous The death probability of cargo transportation accidents has increased by 32.40%, while the impact of other sub-variables such as punctured tires, brake failures and engine fires on the severity of hazardous materials transportation accidents is not significant.

(2) Traffic accidents involving hazardous materials transportation vehicles have increased the probability of fatal accidents by more than three times. Among the hazardous materials transportation accidents, accidents involving hazardous materials vehicles as the main responsible party account for more than 80%. Among them, the probability of fatal accidents in tankers, oil tankers and trucks in traffic accidents increased by 213.40%, 287.60% and 23.30%, respectively. In total, 80% of the deaths of hazardous materials are closely related to tankers (tankers and oil tankers), which can be used to strictly monitor the safety of tanker drivers.

(3) The probability of death of hazardous materials in rainy and snowy weather has increased by 155.20% compared to other weather types, and it is avoided as much as possible in the choice of driving itinerary; similarly, the choice of night without lighting roads makes occurrence probability of the death of hazardous materials has increased by 134.6%, and drivers should try to reduce their travel time at night. Compared with other quarters, the probability of fatal accidents during the second quarter (summer) of hazardous materials transportation vehicles increased by 39.50%. The results show that accidents are closely related to weather temperature.

(4) In terms of space characteristics, the probability of fatal accidents in hazardous materials transportation increased by 81.10%, 42.60%, 31.60%, and 13.20% for highways, national roads, provincial roads, and urban roads (intersections); when transportation vehicles are in East China, once a traffic accident occurs, the probability of fatal accidents is significantly higher than in other regions.

### 6.2. Suggestions 

According to the above research results, in order to effectively mitigate or avoid the occurrence of hazardous materials transportation accidents, it is necessary to take some considerable measures from the perspective of drivers or driving choice behavior to avoid the emergence of factors that have a significant impact on the severity of hazardous materials transportation accidents. Specific suggestions [28,29,30,31,32] are as follows.

(1) Hazardous materials vehicles are often overloaded in the course of transportation. In order to avoid this phenomenon, a load-bearing detection device can be installed in the load-bearing inspection site of hazardous materials transportation vehicles. When carrying a load, the detection device will notify relevant personnel to load within a reasonable vehicle load range through detection, display and voice reminders to avoid overloading and causing hazardous materials transportation accidents during transportation. Second, safe transportation is inseparable from the driver’s mental state and safe driving operation behavior. A driver detection device is added to the in-vehicle system to detect the driving time and mental state of the driver in real time (such as the angle of head up and the angle of eye opening, sitting status, etc.) and driver’s operating behaviors (such as the number of times the driver steps on the brakes, the accelerator, the angle of the steering wheel, whether to talk to the escort, answer the phone, listen to songs, etc.) If there are similar unsafe behaviors mentioned above, timely reminders will be made according to the detection results of the system, and cyclic detection is needed to ensure that the previous unsafe behaviors have been corrected.

(2) According to the research results, quarter, weather conditions, and time of the day have a significant impact on the severity of hazardous materials transportation accidents. Therefore, develop a system that can comprehensively consider the above factors and predict the risk of transportation accidents under the above-mentioned combined conditions. Before the departure of the transportation, analyze the safety and reliability of this transportation through the system to avoid high transportation as much as possible. The emergence of an accident risk combination environment reduces the occurrence of hazardous materials transportation accidents.

(3) The number of hazardous materials transportation accidents with different road types and geographical distributions is quite different. According to research results, for example, the risk of hazardous materials transportation accidents on highways in East China is higher than other roads and areas. In view of this, for the high-incidence areas of transport accidents, relevant departments can implement special policies for hazardous materials vehicles, such as lower speed limits, requiring the selection of roads with better road conditions, and the choice of routes to avoid residents as much as possible. There are many areas, reasonable adjustment of the traffic zone in the city, more slogans and signs on the road to remind the drivers of hazardous materials and so on, so as to actively avoid the occurrence of hazardous materials transportation accidents.

### 6.3. Future Study

However, several limitations still exist in our work, and future work will focus on two aspects:

(1) Based on the results of the findings of hazardous materials transportation accidents, the risk prediction model of hazardous materials road traffic accidents will be established, which is to predict and analyze the risk of freight traffic accidents under the mixed traffic conditions, and then improve the road infrastructure and prevent accidents in advance to reduce the occurrence of accidents and casualties.

(2) From the perspective of road safety, in the future, we will study the decision-making of hazardous materials transportation accident risk behavior, and seeks for the relationship between the behavior of hazardous materials participants and accident risk. Next, we will study specific accident prevention measures and control countermeasures under different scenarios.

## Figures and Tables

**Table 1:** Independent variables of severity of hazardous materials transportation accident.

Variable Category	Variable Name	Variable Description
Driver properties	Violation	1: illegal carriage, 2: overload, 3: fatigue driving
	Unsafe driving behavior	1: Failure to maintain a safe distance; 2. Improper operation; 3. Excessive speed
	Accident liability	1:hazardous materials vehicles, 2:other vehicles
Vehicle properties	Packaging problem	1: valve failure; 2: broken tank; 3: tank fire
	Vehicle problem	1:flat tire, 2:vehicle control, 3: brake failure, 4:engine fire, 5:other causes, 6: other vehicle causes
	Model category	1: tank truck, 2:oil tank truck, 3: illegally modified vehicle, 4: truck
Environmental properties	weather	1: sunny, 2: cloudy, 3: rainy, 4: haze
	Lighting (time)	1: daytime, 2: night lighting, 3: night without lighting
	Month distribution	1: first quarter, 2: second quarter, 3: third quarter, 4: fourth quarter
Road properties	Road grade	1: expressway, 2: national highway, 3: provincial highway, 4: county road and below, 5: urban road(intersection)
	Regional distribution	1: North China, 2: East China, 3: South China, 4: Central China, 5: Northwest China, 6: Southwest China, 7: Northeast China

**Table 2:** Analysis of factors influencing the severity of hazardous materials transportation accidents.

Variable	Deaths	Injuries	Property Damage	Z Value	*p* Value
**Illegal Behavior**				−1.328	<0.001
1. Illegal carriage	3(15.00%)	6(30.00%)	11(55.00%)		
2. The overload	5(31.25%)	4(25.00%)	7(43.75%)		
3. Fatigue driving	7(17.95%)	11(28.21%)	21(53.85%)		
**Unsafe Driving Behavior**				−0.218	<0.001
1. Failure to maintain a safe distance	10(10.42%)	21(21.88%)	65(67.71%)		
2. Improper operation	3(4.29%)	18(25.71%)	49(70.00%)		
3. Excessive speed	13(15.66%)	23(27.71%)	47(56.63%)		
**Accident Liability**				−1.528	<0.001
1. Hazardous materials vehicle	36(8.76%)	91(22.14%)	284(69.10%)		
2. Other vehicles	9(9.47%)	24(25.26%)	62(65.26%)		
**Packing Problem**				−0.443	<0.001
1. Valve failure	1(4.76%)	2(9.52%)	18(85.71%)		
2. Broken tank	0(0.00%)	1(3.85%)	25(96.15%)		
3. Tank fire	2(16.67%)	3(25.00%)	7(58.33%)		
**Vehicle Problem**				−0.225	<0.001
1. Flat tire	0(0.00%)	1(5.56%)	17(94.44%)		
2. Loss control	3(20.00%)	5(33.33%)	7(46.67%)		
3. Brake failed	0(0.00%)	1(16.67%)	5(83.33%)		
4. Engine fire	0(0.00%)	0(0.00%)	2(100.00%)		
5. Other	0(0.00%)	2(33.33%)	4(66.67%)		
6. Other vehicle problem	0(0.00%)	1(14.29%)	6(85.71%)		
**Vehicle Category**				−1.091	<0.001
1. Tank truck	15(6.38%)	53(22.55%)	167(71.06%)		
2. Oil tank truck	18(10.29%)	40(22.86%)	117(66.86%)		
3. Illegally modified vehicle	0(0.00%)	0(0.00%)	5(100.00%)		
4. Truck	9(13.64%)	18(27.27%)	39(59.09%)		
**Weather**				−1.964	<0.001
1. Sunny	16(14.04%)	25(21.93%)	73(64.04%)		
2. Cloudy	8(4.52%)	37(20.90%)	132(74.58%)		
3. Rainy	19(9.45%)	50(24.88%)	132(65.67%)		
4. Haze	2(18.18%)	3(27.27%)	6(54.55%)		
**Lighting**				−0.655	<0.001
1. Daytime	24(7.57%)	70(22.08%)	223(70.35%)		
2. Night lighting	2(6.90%)	4(13.79%)	23(79.31%)		
3. Night without lighting	18(12.95%)	35(25.18%)	86(61.87%)		
**Month Distribution**				−0.655	<0.001
1. First quarter	8(8.51%)	21(22.34%)	65(69.15%)		
2. Second quarter	16(10.39%)	35(22.73%)	103(66.88%)		
3. Third quarter	13(9.03%)	32(22.22%)	99(68.75%)		
4. Fourth quarter	11(9.09%)	31(25.62%)	79(65.29%)		
**Road Grade**				−1.091	<0.001
1. Expressway	23(10.45%)	56(25.45%)	141(64.09%)		
2. National highway	8(8.51%)	21(22.34%)	65(69.15%)		
3. Provincial highway	8(13.33%)	15(25.00%)	37(61.67%)		
4. County road and below	1(3.70%)	9(33.33%)	17(62.96%)		
5. Urban road(intersection)	5(5.68%)	14(15.91%)	69(78.41%)		
**Regional Distribution**					
1. North China	7(12.28%)	12(21.05%)	38(66.67%)		
2. East China	18(9.73%)	43(23.24%)	124(67.03%)		
3. South China	3(5.66%)	13(24.53%)	37(69.81%)		
4. Central China	3(4.92%)	15(24.59%)	43(70.49%)		
5. Northwest China	7(10.29%)	14(20.59%)	47(69.12%)		
6. Southwest China	4(9.52%)	12(28.57%)	26(61.90%)		
7. Northeast China	0(0.00%)	3(16.67%)	15(83.33%)		

**Table 3:** Analysis results of ordered multiple classification Logistic regression.

Influencing Factors	B	S.E.	Wald *χ*^2^	OR	OR Value of 95% of CI	*p* Value
Constant term						
Deaths	−1.461	0.539	9.624	0.23	0.09–0.63	0.000
Injuries	1.790	0.524	7.501	0.94	0.58–1.41	0.001
Property loss (control)	— —	—	— —	—	— — — —	— —
Illegal behavior						
Overload	−1.384	0.513	0.648	1.41	0.57–1.54	0.039
Fatigue driving	−1.934	0.736	1.370	1.20	0.78–1.95	0.018
Illegal carriage (control)	— —	—	— —	—	— — — —	— —
Unsafe driving behavior						
Failure to maintain a safe distance	−1.435	0.166	4.740	1.647	1.19–2.38	0.036
Expressive speed	−1.236	0.387	1.324	1.539	0.94–2.42	0.025
Improper operation (control)	— —	—	— —	—	— — — —	— —
Accident liability						
Hazardous materials vehicles	−2.429	0.307	12.814	2.363	1.51–2.97	0.019
Other vehicles (control)	— —	— —	— —	— —	— — — —	— —
Packing problem						
Valve failure	−0.597	0.073	1.524	0.302	0.08–0.62	0.374
Broken tank	−0.798	0.175	0.493	0.450	0.11–0.89	0.558
Tank fire (control)	— —	— —	— —	— —	— — — —	— —
Vehicle Problem						
Flat tire	−1.202	0.339	1.393	1.095	0.52–1.78	0.443
Loss control	−1.096	0.128	2.470	1.201	0.63–1.92	0.007
Brake failed	−0.277	0.076	0.467	0.478	0.13–0.96	0.231
Engine fire	−0.176	0.277	0.394	0.166	0.02–0.41	0.209
Other	−0.576	0.292	0.612	0.892	0.34–1.57	0.345
Other vehicle problem (control)	— —	— —	— —	— —	— — — —	— —
Vehicle category						
Tank truck	−1611	0.148	8.166	4.942	2.22–7.30	0.000
Oil tank truck	−1.590	0.204	7.791	3.507	1.94–6.79	0.000
truck	3.888	0.314	5.468	1.848	0.636–3.50	0.027
Illegally modified vehicle (control)	— —	— —	— —	— —	— — — —	— —
Weather						
Sunny	1.370	0.351	4.302	0.733	0.23–1.59	0.778
Cloudy	1.813	0.342	6.099	1.393	0.64–2.11	0.046
Rainy	−2.346	0.445	9.787	2.622	1.28–4.09	0.003
Haze (control)	— —	—	— —	—	— — — —	— —
Lighting						
Night lighting	−0.751	0.212	0.452	1.707	0.92–3.12	0.304
Night without lighting	−1.008	0.588	4.357	1.111	0.44–1.89	0.032
Daytime (control)	— —	— —	— —	— —	— — — —	— —
Month Distribution						
First quarter	−0.871	0.177	0.366	1.174	0.47–1.64	0.328
Second quarter	−0.563	0.242	2.259	2.399	1.51–3.35	0.027
Third quarter	−0.572	0.357	1.829	1.978	1.26–2.77	0.079
Fourth quarter (control)	— —	— —	— —	— —	— — — —	— —
Road Grade						
Expressway	−0.451	0.441	8.759	2.619	1.55–3.78	0.049
National highway	−0.871	0.328	4.588	1.538	0.76–2.31	0.036
Provincial highway	−1.741	0.256	4.389	1.174	0.69–1.84	0.022
Urban road(intersection)	−0.696	0.339	2.373	1.068	0.48–1.78	0.008
County road and below (control)	— —	— —	— —	— —	— — — —	— —
Regional Distribution						
North China	−1.473	0.502	2.619	1.52	0.88–2.24	0.002
East China	−0.473	0.354	8.594	2.21	1.53–2.89	0.041
South China	−0.798	0.305	1.679	1.14	0.56–1.75	0.626
Central China	−1.368	0.399	1.829	1.28	0.61–1.92	0.791
Northwest China	−1.161	0.287	2.581	1.60	0.88–2.32	0.039
Southwest China	−1.368	0.292	1.466	1.09	0.45–1.71	0.550
Northeast China (control)	— —	— —	— —	— —	— — — —	— —

**Table 4:** Elastic analysis of factors that significantly influence the severity of road hazardous materials transportation accidents.

Variable	Pseudo-Elasticity		
	Deaths	Injuries	Property Loss
Overload	44%	2.3%	−21.2%
Fatigue driving	113.8%	−10.5%	−50.8%
Failure to maintain a safe distance	256.8%	−36.7%	−83.1%
Expressive speed	269.3%	−35.7%	−81.4%
Hazardous materials vehicles	323.4%	45.7%	87.3%
Loss control	32.4%	−0.5%	−24.6%
Tank truck	213.4%	−23.5%	−71.1%
Oil tank truck	287.6%	−26.2%	−84.3%
Truck	23.3%	−1.9%	−16.0%
Cloudy	31.7%	−0.6%	−23.7%
Rainy	155.2%	−23.0%	−77.2%
Night without lighting	134.6%	−27.1%	−73.2%
Second quarter	39.5%	−0.7%	−26.4%
Expressway	81.1%	−17.4%	−34.2%
National highway	42.6%	−2.1%	−33.3%
Provincial highway	31.6%	−0.3%	−21.7%
Urban road(intersection)	13.2%	−0.5%	−9.7%
North China	21.9%	−3.3%	−14.1%
East China	114.3%	−28.5%	−52.9%
Northwest China	26.6%	−3.1%	−12.2%

**Table 5:** Proportion of traffic accidents with weather sub-independent variables.

Weather	Death	Injury	Property Loss
Sunny	35.56%	21.74%	21.28%
Cloudy	17.78%	32.17%	38.48%
Rainy and Snowy	42.22%	43.48%	38.48%
Haze	4.44%	2.61%	1.75%
